# Four New *Pinnularia* Ehrenberg Species (Bacillariophyta) from Urban Freshwater Streams in South Korea

**DOI:** 10.3390/plants14203206

**Published:** 2025-10-18

**Authors:** Yuyao Li, Weihan Wang, Byeong-Hun Han, Su-Ok Hwang, Baik-Ho Kim

**Affiliations:** 1 Department of Environmental Science, Hanyang University, Seoul 04763, Republic of Korea; liyuyao7773@163.com (Y.L.); wwhann4811@gmail.com (W.W.); tttyy3@naver.com (B.-H.H.); 2 Dongmoon ENT, Guro-gu, Seoul 08377, Republic of Korea; 3 Research Institute for Natural Sciences, Hanyang University, Seoul 04763, Republic of Korea; spring3974@naver.com; 4 Department of Life Science, Hanyang University, Seoul 04763, Republic of Korea

**Keywords:** epilithic diatoms, integrative taxonomy, novel species, urban freshwater ecosystems, morphology and ultrastructure, molecular phylogeny, cryptic diversity

## Abstract

This study reports the discovery of four novel *Pinnularia* species—*P. latocentra* sp. nov., *P. rhombocentra* sp. nov., *P. seouloflexuosa* sp. nov., and *P. paristriata* sp. nov.—from urban freshwater streams in South Korea. Species delimitation was achieved using a polyphasic approach that integrated light and scanning electron microscopy, ecological profiling, and molecular evidence from SSU rRNA and *rbcL* sequences. Each taxon was confirmed as morphologically and genetically distinct from its closest congeners. Our findings broaden the recognized diversity of *Pinnularia* in East Asia and demonstrate that urban streams, often regarded as degraded habitats, can harbor hidden diatom diversity and ecological complexity. By clarifying diagnostic traits, validating type material in a recognized repository, and aligning molecular and morphological evidence, this study contributes to a more robust taxonomy of *Pinnularia*. These results also highlight the importance of polyphasic taxonomy and the strategic inclusion of urban habitats in diatomological surveys and biodiversity assessments.

## 1. Introduction

Diatoms (Bacillariophyta) represent one of the most diverse and ecologically significant algal lineages, with over 100,000 estimated species worldwide. They are key contributors to global primary production, silica cycling, and climate regulation, while their siliceous frustules provide a robust foundation for taxonomy, bioassessment, and paleoenvironmental reconstruction [1,2,3,4,5]. Their short generation times and sensitivity to physicochemical gradients make them indispensable bioindicators in both lentic and lotic ecosystems [6,7,8,9].

Among diatoms, the genus *Pinnularia* Ehrenberg is especially noteworthy for its global distribution, ecological versatility, and long-standing taxonomic complexity. According to AlgaeBase [5], the genus currently comprises about 880 accepted species, though many additional names have been historically proposed [10]. Importantly, *Pinnularia* species are not confined to freshwater habitats; they also occur in marine and terrestrial environments [11]. They inhabit diverse climates and substrate types, ranging from glacial meltwaters to highly eutrophic or anthropogenically disturbed rivers [12,13,14]. Some taxa, such as *P. borealis* and *P. viridiformis*, are cosmopolitan, while others are restricted to acidic, high-altitude, or polar systems [15,16,17]. This combination of broad distribution and ecological specialization has complicated species delimitation, particularly in the absence of integrative approaches [18].

In South Korea, diatom research has historically emphasized alpine wetlands, reservoirs, and forested mountain streams, yielding records of approximately 40–60 *Pinnularia* taxa [19,20,21]. However, these estimates likely underrepresent actual diversity, because urban and peri-urban streams remain comparatively underexplored despite Korea’s dense urbanization and hydrological modification of major watersheds such as the Han, Nakdong, and Yeongsangang Rivers. Urban streams, subject to nutrient enrichment, conductivity shifts, and sedimentation from impervious runoff and weir regulation, may act as both ecological filters and evolutionary hotspots, fostering overlooked or specialized diatom lineages [22,23,24].

Recent methodological advances have facilitated more rigorous taxonomic resolution. Multi-gene phylogenies (e.g., SSU, *rbcL*) have revealed substantial cryptic diversity within *Pinnularia* [25,26,27], while scanning electron microscopy (SEM) of valve ultrastructure has refined species-level boundaries and enabled critical reassessment of type material [28,29,30]. Experimental studies on physiology, such as freezing tolerance, and comparative analyses across polar, alpine, and temperate habitats have further underscored the ecological breadth of the genus [3,31]. Together, these approaches highlight the necessity of a polyphasic framework that integrates morphology, molecular data, and ecology in *Pinnularia* taxonomy [4,32,33,34].

Against this background, the present study describes four novel *Pinnularia* species—*P. latocentra* sp. nov., *P. rhombocentra* sp. nov., *P. seouloflexuosa* sp. nov., and *P. paristriata* sp. nov.—based on monoclonal strains isolated from urban freshwater streams across South Korea. By combining LM, SEM, molecular markers (SSU, *rbcL*), and ecological profiling, we aim to contribute not only to the refinement of regional taxonomic inventories but also to a broader understanding of how anthropogenically influenced habitats sustain hidden diatom diversity and inform bioindicator development.

## 2. Results

**Figure 1:**
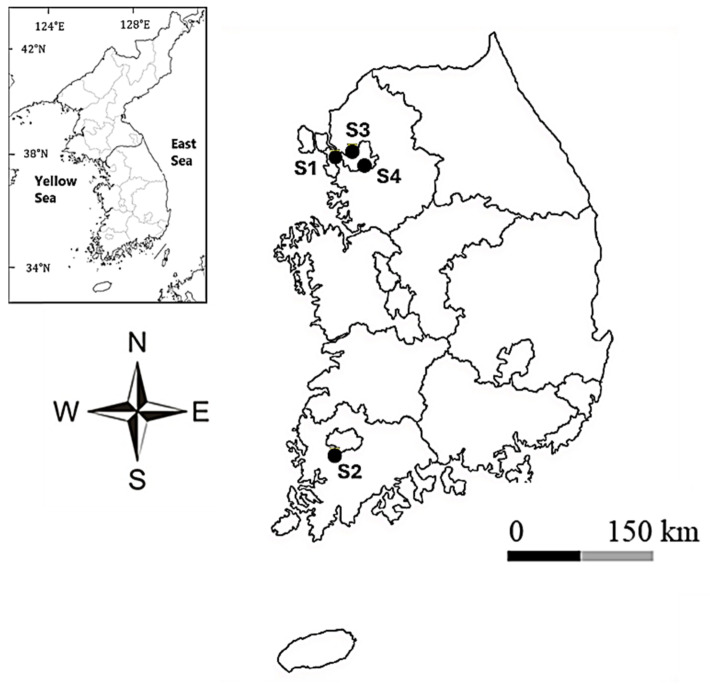
Geographic locations of the four urban freshwater stream sampling sites in South Korea where the *Pinnularia* strains were isolated. S1 = Wonjokssan (Seoul), S2 = Yeongsangang River (Gwangju), S3 = Hongjecheon (Seoul), S4 = Yangjaecheon (Seoul).

This section presents the morphological, ecological, and phylogenetic characteristics of four novel species of the genus *Pinnularia*—*P. latocentra* sp. nov., *P. rhombocentra* sp. nov., *P. seouloflexuosa* sp. nov., and *P. paristriata* sp. nov.—isolated from urban and peri-urban freshwater streams across the Korean Peninsula (Figure 1). Detailed observations were conducted using light microscopy (LM) and scanning electron microscopy (SEM), and molecular analyses were based on the SSU and *rbcL* gene sequences. Diagnostic morphological traits, ecological preferences, and phylogenetic positions were provided for each taxon, along with comparisons with morphologically similar species.

For clarity, we note that GenBank accession numbers (*rbcL*, SSU rDNA) represent DNA sequence records registered in NCBI (National Center for Biotechnology Information, USA). Deposition Numbers (DN) indicate type materials—permanent slides and living cultures—preserved at KCTC (Korean Collection for Type Cultures, KRIBB, Jeongeup, Korea). Holotypes are designated as permanent slides and cultures at KCTC; isotypes are duplicate slides and cultures derived from the same original strains, also preserved at KCTC. Ex-type strains (living cultures corresponding to type material) are maintained at KCTC under the same accession numbers. Laboratory duplicates of all strains are preserved at Hanyang University (HYU, Seoul, Korea), but these are not name-bearing types.

A summary of the site-specific environmental conditions, GenBank accession numbers, and type material deposition is presented in Table 1.

### 2.1. Pinnularia latocentra *Y. Li & B.H. Kim, sp. nov.*

Diagnosis: Valves linear to linear-elliptical with nearly parallel sides, slightly concave at the mid-region, and broadly rounded apices. Length 29.3–31.3 µm, width 5.55–6.76 µm (wild and cultured material). Axial area narrowly lanceolate, expanding asymmetrically into a broad fascia. Striae finely punctate, weakly radiate at the center and slightly convergent toward the apices, 14–16 in 10 µm.

Description (Table 2).

**Figure 2:**
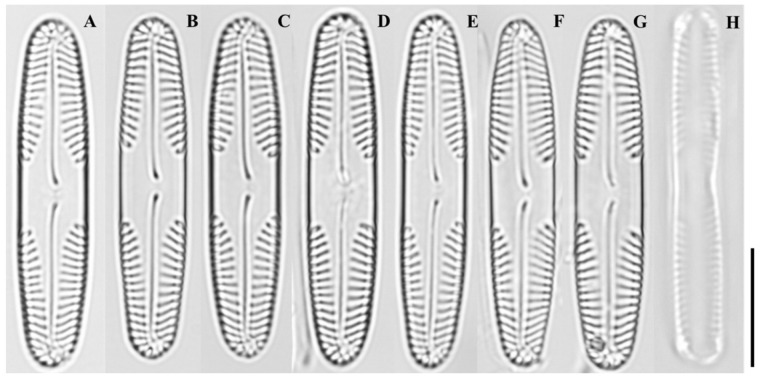
Light micrographs of *Pinnularia latocentra* sp. nov. (**A**–**E**) Valves showing linear to linear-elliptical outlines with broad, asymmetrical central fascia and slightly concave sides; (**F**–**H**) Larger valves with nearly parallel margins, broadly rounded apices, and finely punctate striae that are weakly radiate at the center and slightly convergent near the ends. Scale bar = 10 µm.

LM (Figure 2A–H): Valves linear to linear-elliptical, with a distinctly broad, asymmetrical central fascia. Mid-regions subtly concave; apices broadly rounded. Striae finely punctate, slightly radiate at the center, weakly convergent toward apices.

**Figure 3:**
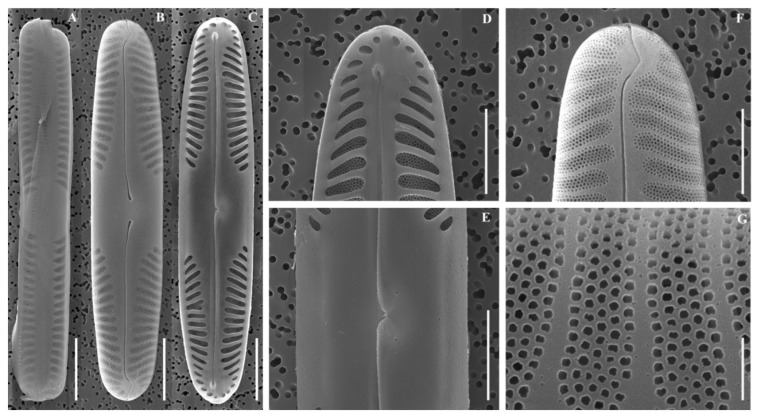
Scanning electron micrographs (SEM) of *Pinnularia latocentra* sp. nov. (**A**) Girdle view; (**B**) External valve view; (**C**) Internal valve view; (**D**) Internal view of valve apices; (**E**) Central area; (**F**) External view of valve apices; (**G**) External detail of an alveolus. Scale bars: (**A**–**F**) = 10 µm; (**G**) = 5 µm.

SEM (Figure 3A–F): Raphe lateral with drop-shaped proximal ends, deflected unilaterally. Terminal fissures hook-shaped externally; raphe internally ending in small helictoglossae. Five alveoli per valve, ghost striae absent.

Type material.

Holotype: KCTC (Korean Collection for Type Cultures, KRIBB, Jeongeup, Korea), permanent slide, accession no. AG61352.

Isotypes: KCTC, duplicate slides and cultures, accession nos. AG61363 (strain WJS230803B5A3) and AG61364 (strain WJS230803B5A4).

Type locality: Hongjecheon, Seoul, South Korea (37°34′03.29″ N, 126°54′57.87″ E).

Etymology: The epithet latocentra derives from the Latin latus (broad) and centrum (center). It refers to the broad, asymmetrical central fascia that is the most characteristic feature of this species, readily distinguishing it from morphologically allied taxa.

Ecology: Found in low-turbidity urban streams with moderate dissolved oxygen (~5.51 mg L^−1^) and neutral to slightly alkaline pH (~6.19).

### 2.2. Pinnularia rhombocentra *Y. Li & B.H. Kim, sp. nov.*

Diagnosis: Valves rhombic-lanceolate with broadly rounded ends. Length 31.4–48.2 µm, width 7.0–9.3 µm. Axial area distinctly rhombic, expanded at the center. Central area narrow and slightly asymmetric. Striae radiate throughout, 16–20 in 10 µm.

Description (Table 3).

**Figure 4:**
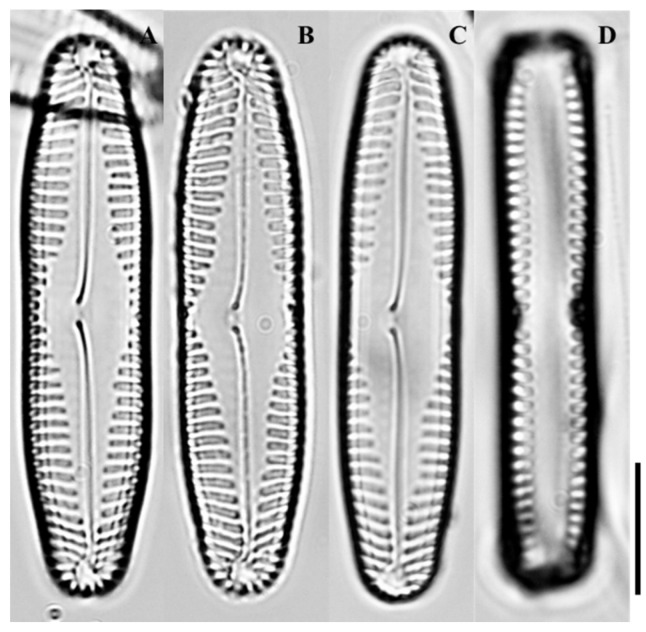
Light micrographs of *Pinnularia rhombocentra* sp. nov. (**A**) Valve in girdle view showing overall outline; (**B**) Valve in external view highlighting the rhombic central area; (**C**) Valve showing radiate striae across the central region; (**D**) Valve apex showing rounded ends and striae arrangement. Scale bar = 10 µm.

LM (Figure 4A–D): Valves rhombic-lanceolate with distinct rhombic central area. Apices broadly rounded. Striae radiate across the valve, denser at apices.

**Figure 5:**
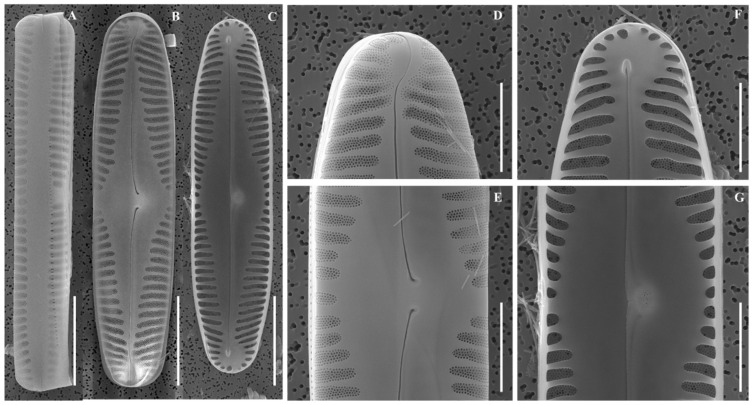
Scanning electron micrographs (SEM) of *Pinnularia rhombocentra* sp. nov. (**A**) Girdle view; (**B**) External valve view; (**C**) Internal valve view; (**D**) External view of valve apices; (**E**) Central area; (**F**) Internal view of valve apices; (**G**) Central area. Scale bars: (**A**–**C**) = 10 µm; (**D**–**G**) = 5 µm.

SEM (Figure 5A–G): Raphe filiform, externally straight with expanded proximal ends. Terminal fissures slightly deflected. Internally, raphe endings with small helictoglossae. Well-defined alveoli visible.

Type material.

Holotype: KCTC, permanent slide, accession no. AG61353.

Isotypes: KCTC, duplicate slides and cultures, accession nos. AG61365 (strain YSG231115B7A1) and AG61366 (strain YSG231115B7A2).

Type locality: Yeongsan-gang, Gwangju, South Korea (35°00′20.71″ N, 126°44′28.47″ E).

Etymology: The epithet rhombocentra combines the Greek rhombos (diamond-shaped) and Latin centrum (center). It highlights the distinctive rhombic outline of the central area, which is a defining character that separates this taxon from other similar *Pinnularia* species.

Ecology: Recorded in moderately turbid rivers with higher conductivity (362 µS cm^−1^) and slightly alkaline pH (~7.8).

### 2.3. Pinnularia seouloflexuosa *Y. Li & B.H. Kim, sp. nov.*

Diagnosis: Valves distinctly flexuous, linear-lanceolate with capitate ends. Length 147–151 µm, width 19–21 µm. Axial area broad, slightly eccentric; central area fascia-like. Striae weakly radiate at center, parallel to slightly convergent near apices, 14–18 in 10 µm.

Description (Table 4).

**Figure 6:**
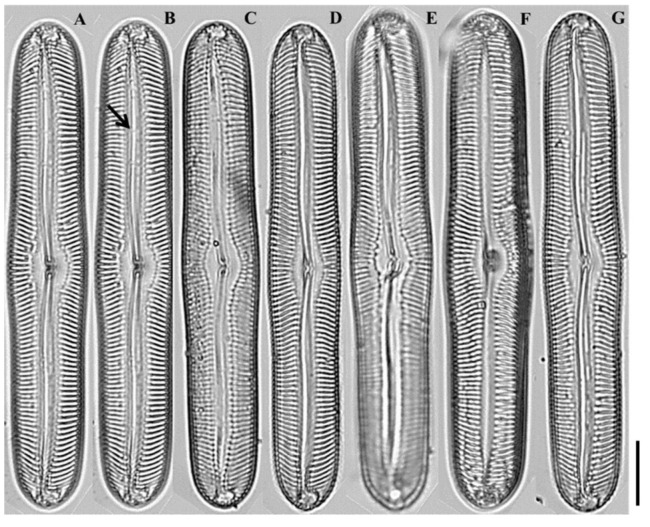
Light micrographs of *Pinnularia seouloflexuosa* sp. nov. (**A**–**C**) Smaller valves showing slightly flexuous linear-lanceolate outlines with narrow central fascia; (**D**–**E**) Medium-sized valves exhibiting more distinct curvature and broader fascia; (**F**–**G**) Larger valves with capitate apices and weakly radiate striae near the center. Scale bar = 20 µm.

LM (Figure 6A–G): Valves linear-lanceolate, distinctly flexuous, apices capitate. Central area fascia-like. Striae weakly radiate at center, convergent near apices.

**Figure 7:**
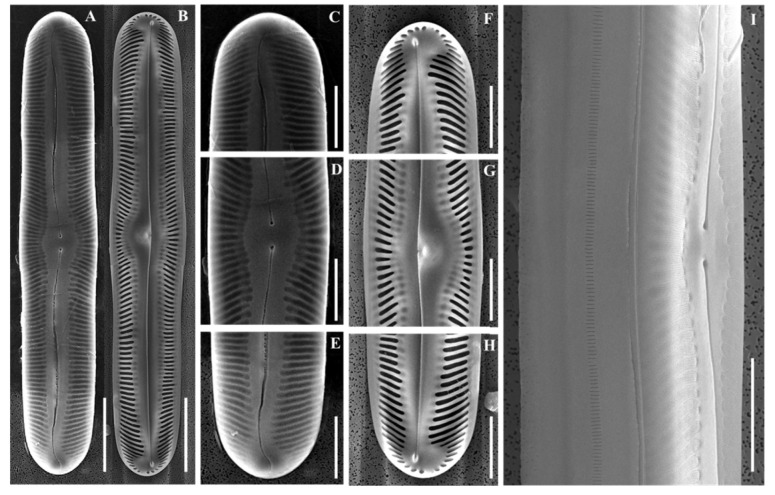
Scanning electron micrographs (SEM) of *Pinnularia seouloflexuosa* sp. nov. (**A**) External and (**B**) internal views of whole valves; (**C**–**E**) External valve details; (**F**–**H**) Internal valve views; (**D**,**G**) Central area close-ups; (**I**) External central area shown in the girdle view. Scale bars: (**A**,**B**) = 20 µm; (**C**–**I**) = 10 µm.

SEM (Figure 7A–I): Raphe filiform, proximal ends externally expanded, deflected laterally. Terminal fissures hook-shaped. Internally ending in small helictoglossae. Striae uniseriate, areolae elongated transapically.

Type material.

Holotype: KCTC, permanent slide, accession no. AG61340.

Isotypes: KCTC, duplicate slides and living cultures, accession nos. AG61343 (strain HJC230531B3A1) and AG61344 (strain HJC230531B3A2).

Type locality: Hongjecheon, Seoul, South Korea (37°34′03.29″ N, 126°54′57.88″ E).

Etymology: The epithet *seouloflexuosa* commemorates the type locality (Seoul) and Latin *flexuosus* (bent, sinuous). It emphasizes the rare flexuous valve outline, a prominent morphological trait within the genus, and reflects its origin in an urban environment.

Ecology: Occurs in moderately warm waters (~25 °C), with very low turbidity (1.2 NTU) and circumneutral pH (~7.6).

### 2.4. Pinnularia paristriata *Y. Li & B.H. Kim, sp. nov.*

Diagnosis: Valves linear-elliptical to narrowly lanceolate, apices subrostrate. Length 57.6–75.1 µm, width 13.8–16.5 µm. Axial area narrow to moderately broad, central area fascia-like. Striae parallel to weakly radiate at center, radiate toward apices, 20–24 in 10 µm.

Description (Table 5).

**Figure 8:**
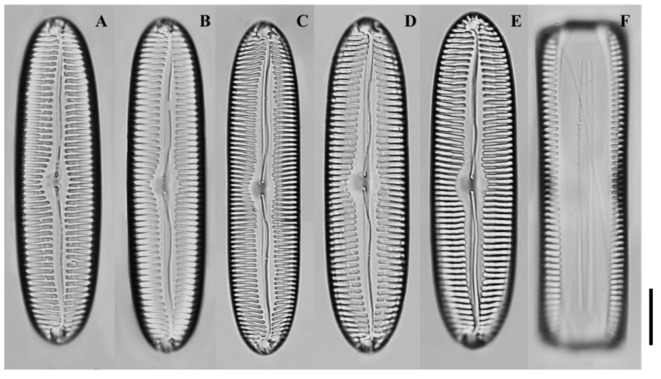
Light micrographs of *Pinnularia paristriata* sp. nov. (**A**–**D**) Valves showing linear-elliptical to narrowly lanceolate outlines with parallel to slightly convex margins and broad central fascia; (**E**–**F**) Larger valves with subrostrate apices and striae that are parallel to weakly radiate at the center and denser toward the ends. Scale bar = 10 µm.

LM (Figure 8A–F): Valves linear-elliptical to lanceolate, apices subrostrate. Central area broad fascia-like. Striae parallel to weakly radiate at center, denser toward apices.

**Figure 9:**
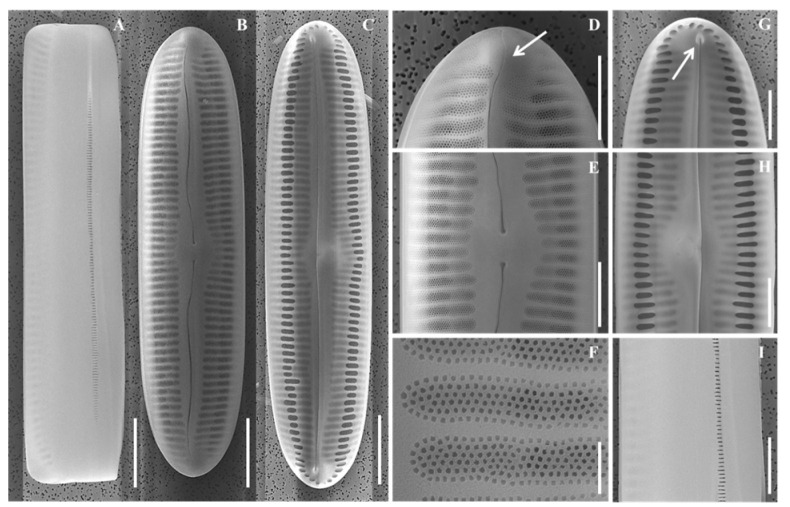
Scanning electron micrographs (SEM) of *Pinnularia paristriata* sp. nov., showing external and internal valve structure, including apices and central area architecture. (**A**) External and (**B**) internal views of whole valves; (**C**–**E**) External valve details; (**F**–**H**) Internal valve views; (**D**,**G**) Central area close-ups; (**I**) External central area shown in the girdle view. Scale bars: (**A**–**C**) = 10 µm; (**D**,**E**, **G**–**I**) = 5 µm; (**F**) = 1 µm.

SEM (Figure 9A–I): Raphe lateral, proximal ends expanded, slightly deflected. Terminal fissures hook-shaped. Internally ending in small helictoglossae. Areolae arranged linearly, striae punctate.

Type material.

Holotype: KCTC, permanent slide, accession no. AG61387.

Isotypes: KCTC, duplicate slides and living cultures, accession nos. AG61390 (strain YJC241112B2A1) and AG61391 (strain YJC241112B2A2).

Type locality: Yangjaecheon, Seoul, South Korea (37°29′23.38″ N, 127°03′52.23″ E).

Etymology: The epithet *paristriata* is derived from Latin *para* (beside, near) and *striata* (striated). It refers to the diagnostic pattern of closely spaced, nearly parallel striae that dominate the valve surface.

Ecology: Found in streams with moderate conductivity (~518 µS cm^−1^), slightly alkaline pH (~8.0), and dissolved oxygen around 11 mg L^−1^.

### 2.5. Phylogenetic Placement

Partial SSU rRNA and *rbcL* sequences were successfully obtained from all four novel *Pinnularia* species. Maximum likelihood (ML) analyses based on both markers consistently resolved each species as a distinct and well-supported lineage that was evidently separated from all previously described congeners. These results provide strong genetic evidence that supports the recognition of each taxon as a novel species.

**Figure 10:**
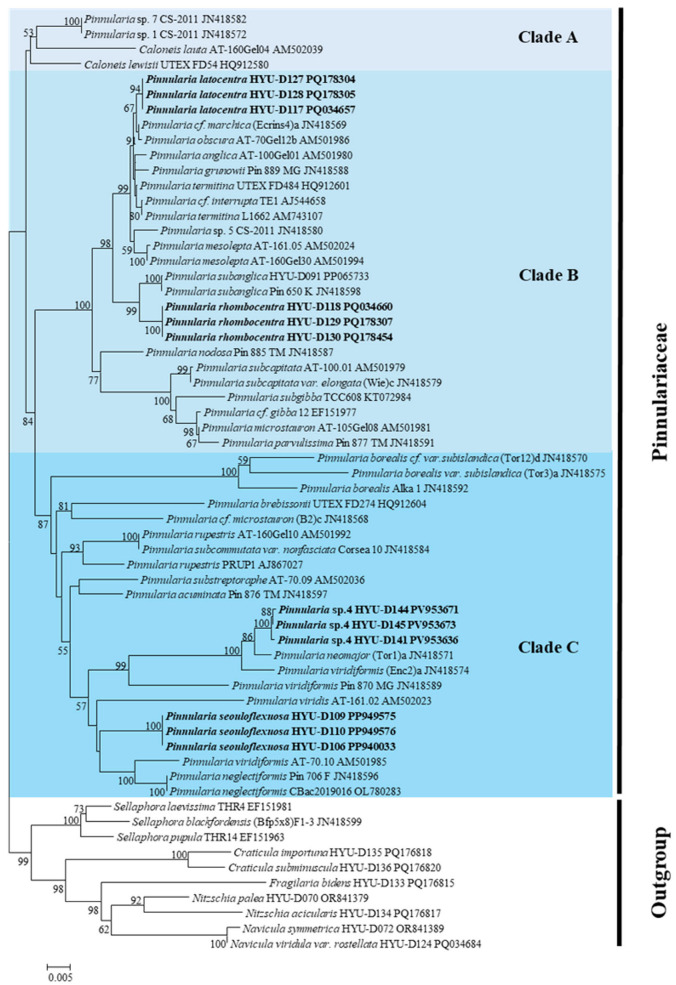
Maximum likelihood (ML) phylogenetic tree based on nuclear 18S small subunit ribosomal RNA (SSU rRNA) sequences, showing the position of *P. latocentra*, *P. rhombocentra*, *P. seouloflexuosa*, and *P. paristriata* among related *Pinnularia* species.

In the SSU rRNA phylogeny (Figure 10), *Pinnularia seouloflexuosa* clustered with *P. neglectiformis* and *P. viridis,* forming a clade with moderate bootstrap support (62%). Despite this proximity, *P. seouloflexuosa* was separated as an independent lineage from *P. neglectiformis* and *P. viridis* with moderate bootstrap support (62%). *P. rhombocentra* was grouped with *P. subanglica* and *P. nodosa*, supported by bootstrap values of 73–79%. Each of these placements indicates that the novel taxa are genetically distinct from their morphologically similar relatives. *P. latocentra* formed a separate, well-supported branch (bootstrap 81%) adjacent to *P. obscura* and *P.* cf. *marchica* but was readily distinguished by its broad asymmetrical fascia and lack of ghost striae, which are features rarely observed in related taxa. *P. paristriata* was placed as an independent basal lineage of the *P. microstauron* complex and *P.* cf. *borealis* with high support (bootstrap, 88%), exhibiting evident differentiation.

To complement the SSU rRNA tree, pairwise evolutionary divergences among the 20 closest *Pinnularia* species were calculated using the Kimura 2-parameter model (Table 6). These values ranged from 0.006 to 0.042 among the new taxa and their nearest relatives, well within the expected interspecific range for diatoms and apparently above the commonly observed intraspecific threshold (<0.005), which is consistent with previous studies establishing molecular divergence thresholds in *Pinnularia* [15,16,40,41,42].

**Figure 11:**
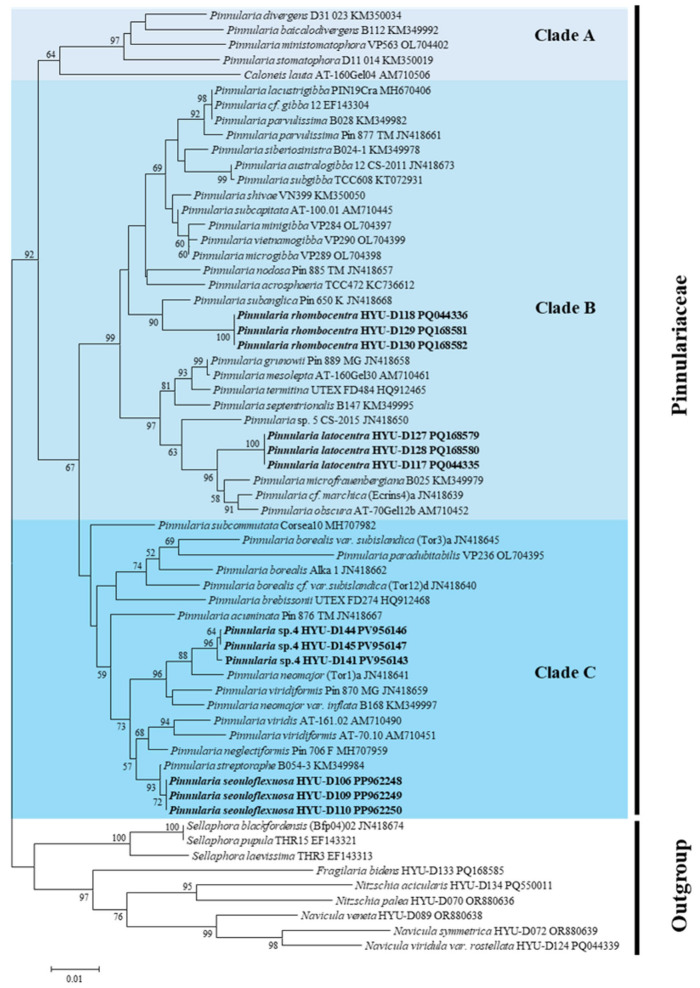
Maximum likelihood (ML) phylogenetic tree based on chloroplast-encoded ribulose-1,5-bisphosphate carboxylase/oxygenase large subunit (*rbcL*) gene sequences, illustrating the placement of *P. latocentra*, *P. rhombocentra*, *P. seouloflexuosa*, and *P. paristriata* within the genus *Pinnularia*.

The *rbcL* phylogeny (Figure 11) was generally consistent with the SSU results, confirming that each new species occupied a distinct evolutionary position: *P. seouloflexuosa* (bootstrap, 78%), *P. rhombocentra* (68%), *P. latocentra* (64%), and *P. paristriata* (84%). Pairwise distances based on the *rbcL* sequences (Table 7) ranged from 0.005 to 0.078. Notably, the smallest observed distance (0.005) was between *P. rhombocentra* and *P. subanglica*, whereas other comparisons exceeded 0.02, supporting robust genetic divergence even within the same clade. Together with the SSU results, these data support species-level differentiation and highlight lineage independence.

Full strain names and GenBank accession numbers used in the phylogenetic analyses are provided in Supplementary Table S1, which includes both novel and comparative taxa used in the SSU and *rbcL* trees. Secondary structure comparisons corresponding to the SSU- and *rbcL*-based clades are shown in Supplementary Figures S1–S4. Table 1 summarizes the strain type information and site-specific environmental conditions of the four novel species described in this study. The consistent correspondence between molecular data, diagnostic morphology, and ecological preferences reinforces the polyphasic taxonomic framework and highlights the previously undescribed diversity within *Pinnularia* in urbanized freshwater habitats.

### 2.6. Distribution and Habitat Preferences

The four novel *Pinnularia* species described in this study exhibit distinct ecological preferences and habitat specializations across multiple freshwater systems in South Korea, comprising urban, peri-urban, and forested tributaries. Their distributions indicate ecological differentiation through niche partitioning and environmental filtering under varying degrees of anthropogenic and natural disturbance.

*P. latocentra* was consistently recorded in clear, low-turbidity streams (~3.3 NTU) with neutral to slightly alkaline pH (~7.2) and moderate dissolved oxygen (~9.4 mg/L). Its broad, asymmetrical central fascia and thickened valve walls suggest an adaptation to stable hydraulic regimes and low sediment abrasion.

*P. rhombocentra* occurred in urban streams subjected to episodic sediment influx with moderate turbidity and conductivity levels. Its rhombic axial area and intermediate striae density (14–15 per 10 µm), combined with distinct alveolation (4–6 alveoli per valve), indicate morphological adjustments to variable flow and sediment regimes.

*P. seouloflexuosa* dominated eutrophic urban streams characterized by high turbidity (>10 NTU), low dissolved oxygen, and elevated conductivity. Its large size (up to 151 µm), flexuous axial area, and high length-to-width ratio (7.01–7.66) demonstrate tolerance to physicochemical stressors and sediment resuspension.

*P. paristriata* was isolated from shaded, forested tributaries with slightly acidic pH (~6.8), low conductivity, and minimal anthropogenic influence. It is distinguished by a wide central fascia and short striae, which indicate an adaptation to low-light, low-flow environments.

Collectively, these four *Pinnularia* species exhibit non-overlapping ecological niches, consistent morphometric adaptation, and evident phylogenetic separation, reinforcing their taxonomic novelty. These findings are concordant with ecological niche partitioning and habitat specialization reported for *Pinnularia* taxa in contrasting acidic, polar, and eutrophic systems [24,26,30], further supporting the robustness of our conclusions.

## 3. Discussion

This study establishes an integrative framework for delimiting four novel *Pinnularia* species—*P. latocentra, P. rhombocentra, P. seouloflexuosa,* and *P. paristriata*—from Korean urban streams. By combining LM/SEM-based morphology, ecological profiling, and molecular markers (SSU, *rbcL*), we highlight the overlooked diversity of this genus in anthropogenically influenced habitats, consistent with findings from underexplored regions [10,12,30,32,36].

Morphological differentiation and taxonomic coherence. Each taxon is morphologically distinct from its closest congeners (Table 2, Table 3, Table 4 and Table 5). *Pinnularia latocentra* differs from *P. microfrauenbergiana* and *P. siberiosinistra* by its broader fascia and absence of ghost striae [12,36]. *P. rhombocentra* is distinguished from members of the P. *parvulissima* complex, such as *P. subanglica* and *P. nodosa*, by its rhombic central area and radiate striae [10,37,43]. *P. seouloflexuosa* exhibits a strongly flexuous outline, unlike the straight or weakly curved valves of *P. streptoraphe* and *P. spinifera* [10,44,45]. *P. paristriata* possesses parallel striae and a narrow axial area, separating it from P. viridis and *P. viridiformis* [34,46]. These differences were observed consistently in both field-collected and cultured material, reinforcing the validity of each species [47].

Molecular corroboration with cautious interpretation. Pairwise divergences in SSU and *rbcL* sequences (Table 6 and Table 7) exceeded typical intraspecific thresholds for diatoms [20,48]. For example, *rbcL* distances between *Pinnularia rhombocentra* and *P. subanglica* exceeded 0.02, consistent with species-level separation [12,36]. The congruence of LM/SEM traits with molecular discontinuities supports their recognition as distinct taxa, in agreement with recent integrative studies in *Pinnularia* [12,20,49]. In accordance with reviewer recommendations, the phylogenetic trees (Figure 10 and Figure 11) are presented solely as supporting evidence for species-level distinctiveness, without making inferences about deeper evolutionary relationships.

Ecological and biogeographic context. The four taxa exhibit non-overlapping ecological associations. *Pinnularia paristriata* was recorded in shaded, oligotrophic tributaries, while *P. seouloflexuosa* occurred in nutrient-rich, high-conductivity channels. *P. latocentra* was associated with low-turbidity, moderately conductive streams, and *P. rhombocentra* occurred in sandy-bottom channels under episodic disturbance. Such niche partitioning parallels patterns reported for specialized *Pinnularia* in polar, alpine, and tropical ecosystems [11,14,41,50]. Urban rivers may therefore function as reservoirs of cryptic diatom diversity, consistent with observations from East Asia [51], studies of pseudocryptic diversity in Europe [52], and recent surveys in the Damavand River basin, Iran [53].

Implications for taxonomy and biomonitoring. This study emphasizes the necessity of a polyphasic approach in *Pinnularia* taxonomy [23,49,54]. Morphological convergence and phenotypic plasticity alone have historically confounded species recognition in this genus [10,36,55]. Our findings further underscore the importance of reassessing historical type specimens for taxonomic clarity, as emphasized by Jahn [56] in the rediscovery of the *Pinnularia* gastrum type specimen. By integrating SEM ultrastructure and molecular markers, we improved diagnostic precision. Furthermore, the habitat fidelity of *P. latocentra* and *P. paristriata* suggests their potential as sensitive indicators of conductivity and sediment changes, complementing existing diatom-based biomonitoring frameworks [6,57].

Synthesis. Recognition of *Pinnularia latocentra, P. rhombocentra, P. seouloflexuosa,* and *P. paristriata* extends the known diversity of *Pinnularia* in East Asia and demonstrates that even heavily modified urban streams can harbor novel diatom lineages. By aligning morphology, molecular evidence, and ecology, this study contributes to a conservative yet robust taxonomy and underscores the ecological importance of urban freshwater habitats in sustaining hidden microbial diversity [30,32,58].

## 4. Materials and Methods

### 4.1. Study Area and Diatom Isolation

Between May 2023 and November 2024, epilithic diatom samples were collected from four urban freshwater streams in South Korea: Wonjokssan (Seoul), Hongjecheon (Seoul), Yangjaecheon (Seoul), and the Yeongsangang River (Gwangju). These sites represent a range of physicochemical conditions and anthropogenic disturbance, from nutrient-enriched urban channels to moderately impacted mid-gradient river segments (Figure 1; Table S1). At each location, submerged cobbles (ca. 5 × 5 cm) were sampled from shallow flowing areas (<0.3 m depth). Epilithic biofilms were gently brushed from cobble surfaces with sterilized soft-bristle brushes and transferred into sterile 200 mL polypropylene containers partially filled with ambient stream water to preserve cell integrity during transport.

On-site physicochemical parameters—including water temperature (WT), pH, turbidity (Turb), dissolved oxygen (DO), and electrical conductivity (EC)—were measured using a portable multiparameter probe (U-50 Series; HORIBA, Kyoto, Japan). These environmental data were used to characterize the ecological niches of each strain. Twelve clonal strains of the four novel *Pinnularia* taxa were successfully isolated by micropipette selection under an inverted microscope (IX73, Olympus, Tokyo, Japan) and maintained in WC medium at 20 °C under a 14:10 h light: dark cycle. These clonal isolates were used for morphological, ecological, and molecular analyses [52,59].

### 4.2. Isolation and Cultivation

Single diatom cells were isolated with fine-tipped glass microcapillaries using an inverted microscope (Eclipse Ts2; Nikon, Tokyo, Japan). Clonal strains were first established in 96-well plates containing Diatom Medium (DM; CCAP formulation) and incubated under controlled conditions (20 °C, 12:12 h light: dark cycle, light intensity 120 µmol m^−2^ s^−1^). After 2–4 weeks of initial growth, cultures were successively transferred to larger volumes (24-well plates, 50 mL flasks). Strains were subcultured every 30–45 days to preserve genetic and physiological stability [60,61].

### 4.3. Morphological and Ultrastructural Examination

For frustule cleaning, dense culture material was digested with a 1:3 (*v*/*v*) mixture of nitric and sulfuric acids at low heat (3–5 min), followed by repeated rinsing with distilled water until neutral pH, following established diatom protocols [62,63]. Cleaned material was mounted in Naphrax^®^ for LM observations using a Nikon Eclipse E600 microscope with a DS-Fi3 digital camera (Nikon Corporation, Tokyo, Japan). For each taxon, at least 60 valves were measured to assess intrapopulation variability. Parameters recorded included valve length, width, central and axial area features, striae and areolar densities, and raphe morphology.

For SEM, frustules were filtered onto 0.2 µm polycarbonate membranes, mounted on aluminum stubs with conductive adhesive, and sputter-coated with platinum. Imaging was conducted with a field-emission SEM (Apreo S, Thermo Fisher Scientific, Waltham, MA, USA) to resolve fine ultrastructural features critical for taxonomic differentiation in *Pinnularia* (e.g., internal distal raphe ends, areolar occlusions, virgae and vimines) [22,59].

### 4.4. DNA Extraction and Phylogenetic Analysis

DNA was extracted from actively growing cultures using the DNeasy Plant Mini Kit (Qiagen, Hilden, Germany) with minor modifications to improve lysis of silica-encased cells. Two loci were targeted: nuclear SSU rRNA and plastid-encoded *rbcL*. PCR was performed with standard primers and conditions (95 °C for 4 min; 35 cycles of 95 °C for 30 s, 56 °C for 30 s, 72 °C for 1 min; final extension 72 °C for 7 min). Products were checked on 1% agarose gels, purified using the QIAquick Gel Extraction Kit (Qiagen GmbH, Hilden, Germany), and sequenced (Bionics, Seoul, Korea). Sequences were assembled, aligned using ClustalW in MEGA7 [64], and analyzed under Maximum Likelihood with 1000 bootstrap replicates using the Kimura 2-parameter model [65], with bootstrap support values calculated following Felsenstein [66].

### 4.5. Taxonomic Validation

Species diagnoses were established under a polyphasic framework integrating LM and SEM morphology, ecological context, and molecular evidence (SSU, *rbcL*). Morphological comparisons were made against published monographs and regional floras [59,61], and nomenclatural validation was cross-checked with AlgaeBase [5] and Diatoms of North America [2]. Type material (holotypes and isotypes) has been deposited in the Korean Collection for Type Cultures (KCTC) with unique accession numbers (Table 1; Supplementary Table S1). This approach improves the resolution of morphologically cryptic diversity and ensures reproducibility in taxonomic practice.

## 5. Conclusions

This study provides a comprehensive taxonomic and ecological characterization of four novel *Pinnularia* species—*P. latocentra*, *P. rhombocentra*, *P. seouloflexuosa*, and *P. paristriata*—discovered in urban streams of South Korea. Species delimitation was achieved using a polyphasic framework that integrated high-resolution LM and SEM morphology, ecological data, and molecular evidence (SSU rRNA, *rbcL*). These combined datasets confirm the distinctiveness and novelty of the four taxa. Our results broaden the recognized diversity of *Pinnularia* in East Asia and demonstrate that urban streams, often regarded as degraded or marginal habitats, can harbor cryptic diatom lineages. This highlights not only the hidden taxonomic diversity but also the frequently overlooked ecological complexity of anthropogenically influenced aquatic systems. By clarifying diagnostic traits, validating type material in a recognized repository, and aligning molecular and morphological data, this work contributes to a more robust taxonomy of *Pinnularia*. Beyond taxonomy, the findings underscore the importance of strategically including urban habitats in diatomological surveys and biomonitoring programs. Such efforts are essential for detecting cryptic species, refining biodiversity assessments, and improving the ecological management of freshwater ecosystems.

## Figures and Tables

**Table 1:** Sampling information, environmental parameters, GenBank accession numbers, and type material deposition for four *Pinnularia* species isolated from urban freshwater streams in South Korea.

Species	Strains	Locations	Date	GPS (Latitude, Longitude)	WT	pH	Turb	DO	EC	*rbcL*	SSU rDNA	DN
*Pinnularia latocentra* sp. nov.	HYU-D117	Wonjokssan	7 July 2023	37°30′21.30″ N, 126°41′29.30″ E	22.1	6.19	10.5	5.51	114	PQ044335	PQ034657	AG61352
	HYU-D127	Wonjokssan	7 July 2023	37°30′21.30″ N, 126°41′29.30″ E	22.1	6.19	10.5	5.51	114	PQ168579	PQ178304	AG61363
	HYU-D128	Wonjokssan	7 July 2023	37°30′21.30″ N, 126°41′29.30″ E	22.1	6.19	10.5	5.51	114	PQ168580	PQ178305	AG61364
*Pinnularia rhombocentra* sp. nov.	HYU-D118	Yeongsangang	15 November 2023	35°00′20.71″ N, 126°44′28.47″ E	13.3	7.84	40.4	11.34	362	PQ044336	PQ034660	AG61353
	HYU-D129	Yeongsangang	15 November 2023	35°00′20.71″ N, 126°44′28.47″ E	13.3	7.84	40.4	11.34	362	PQ168581	PQ178307	AG61365
	HYU-D130	Yeongsangang	15 November 2023	35°00′20.71″ N, 126°44′28.47″ E	13.3	7.84	40.4	11.34	362	PQ168582	PQ178454	AG61366
*Pinnularia seouloflexuosa* sp. nov.	HYU-D106	Hongjecheon	31 May 2023	37°34′03.29″ N, 126°54′57.88″ E	25.1	7.58	1.22	8.36	211	PP962248	PP940033	AG61340
	HYU-D109	Hongjecheon	31 May 2023	37°34′03.29″ N, 126°54′57.88″ E	25.1	7.58	1.22	8.36	211	PP962249	PP949575	AG61343
	HYU-D110	Hongjecheon	31 May 2023	37°34′03.29″ N, 126°54′57.88″ E	25.1	7.58	1.22	8.36	211	PP962250	PP949576	AG61344
*Pinnularia paristriata* sp. nov.	HYU-D141	Yangjaecheon	12 November 2024	37°29′23.38″ N, 127°03′52.23″ E	17.8	7.98	6.12	11.16	518	PV956143	PV953636	AG61387
	HYU-D144	Yangjaecheon	12 November 2024	37°29′23.38″ N, 127°03′52.23″ E	17.8	7.98	6.12	11.16	518	PV956146	PV953671	AG61390
	HYU-D145	Yangjaecheon	12 November 2024	37°29′23.38″ N, 127°03′52.23″ E	17.8	7.98	6.12	11.16	518	PV956147	PV953673	AG61391

GPS: Global Positioning System (latitude, longitude in decimal degrees). WT: Water Temperature (°C). Turb: Turbidity (NTU, Nephelometric Turbidity Units). DO: Dissolved Oxygen (mg L^−1^). EC: Electrical Conductivity (µS cm^−1^). *rbcL*: Ribulose-1,5-bisphosphate carboxylase/oxygenase large subunit gene. SSU rDNA: Small subunit ribosomal DNA. DN: Deposition Numbers assigned to type materials (holotypes, isotypes, ex-type strains) preserved at KCTC (Korean Collection for Type Cultures, KRIBB, Jeongeup, Korea).

**Table 2:** Comparative morphological characteristics of *Pinnularia latocentra* sp. nov. and morphologically similar species based on valve structure and striae patterns.

Characters	*P. latocentra* sp. nov.	*P. microfrauenbergiana*	*P. siberiosinistra*	*P. vietnamogibba*	*P. obscura*
Length (µm)	29.3–31.3	20–25	25–29	34–54	19–36
Width (µm)	5.55–6.76	4.5–5	5	7–8.5	5–6.8
Length/Width Ratio	4.39–5.53	Not documented	Not documented	4.7–6.75	Not documented
Striae density (/10 µm)	14–15	14–15	12–14	10–11	10–16
Alveolus	5, distinct trait	Not documented	Not documented	5, present	Not documented
Valve outline	Linear to linear-elliptical, slightly concave or parallel sides	Narrowly elliptical, convex sides	Narrowly elliptical	Linear to linear-elliptical, slight convexity	linear-lanceolate
Apices (ends)	Broadly rounded	Rounded	Subcapitate	Broadly rounded	cuneiform apices
Striae Pattern	Slightly radiate at center, convergent at ends	Radiate at center, convergent at ends	Strongly radiate at center, convergent at ends	Radiate in middle, strongly convergent at ends	somewhat radiate in the center and convergent at the apices
Raphe	Weakly lateral, filiform	Weakly lateral, filiform	Straight, filiform, weakly noticeable	Weakly lateral	filiform
Axial area	Narrow lanceolate, widens toward central area	Narrow lanceolate, widens toward central area	Narrow lanceolate, slight widening	Moderately broad, ~1/4 of valve width	narrow and linear
Central area	Broad, slightly asymmetric fascia	Wide transverse fascia	Wide transverse fascia	Large, rhombic with a broad fascia	Wide transverse fascia
Ghost striae	None	Not documented	Not documented	Unequal, irregular	Not documented
Proximal raphe ends	Drop-like, deflected to one side, opposite of terminal ends	Drop-like, deflected same as terminal ends	Drop-like, deflected same as terminal ends	Slight unilateral deflection, drop-shaped ends	Small, teardrop-shaped
Terminal raphe fissures	Hooked externally, unilaterally deflected	Hooked externally, unilaterally deflected	Hooked externally, unilaterally deflected	Sickle-shaped, extending to valve margin	Slightly hooked
Raphe branches	Straight, terminate on small helictoglossae	End in simple polar helictoglossae	End in simple polar helictoglossae	Straight, terminate on small helictoglossae	Not documented
References	This study	Kulikovskiy et al. [8]	Kulikovskiy et al. [8]	Kulikovskiy et al. [8]; Kezlya et al. [29]	Krammer [6]; Ciniglia et al. [24]

**Table 3:** Diagnostic morphological features of *Pinnularia rhombocentra* sp. nov. compared with closely related taxa from the *P. parvulissima* complex.

Characters	*P. rhombocentra* sp. nov.	*P. parvulissima*	*P. meridiana*	*P. subanglica*	*P. nodosa*
Length (µm)	39.51–47.23	40–88	34–54	27.9–39.9	40–85
Width (µm)	9.29–11.22	9–12	7–8.5	6.3–7.7	10–13
Length/Width ratio	3.63–5.08	4.5–6.5	Not documented	Not documented	3.5–6
Striae density (/10 µm)	14–15	9–10	10–11	11–14	8–10
Alveolus	4–6, distinct feature	Not documented	Not documented	Not documented	Not documented
Valve outline	Linear to linear-elliptical, slightly concave or parallel sides	Linear, slightly convex sides	Linear-elliptic, parallel to slightly convex sides	Linear, straight to weakly convex, biundulate	Linear, slightly convex sides
Apices (ends)	Broadly rounded	Broadly rostrate to subcapitate	Slightly rostrate to rounded	Capitate, subcapitate in shorter cells	Rounded to subrostrate
Striae pattern	Slightly radiate in center, slightly convergent near apices	Radiate in middle, slightly convergent at ends	Not documented	Radiate in center, convergent near apices	Radiate, slightly convergent near ends
Raphe	Weakly lateral, filiform	Straight, filiform	Lightly lateral, indistinct terminal fissures	Lateral, filiform	Lateral, filiform
Axial area	Rhombic, lanceolate, widens toward center, ~1/2 valve width	Narrowly lanceolate, widens toward center	Moderate width, ~1/4 of valve	Narrowly lanceolate, slight widening near center	Narrowly lanceolate
Central area	No fascia, or small asymmetric fascia	Wide transverse fascia	None	Rhombic, broad fascia	Broad, elliptical
Ghost striae	None	Present	Not documented	Not documented	Not documented
Proximal raphe ends	Drop-like and deflected to the same side but in the opposite direction of the terminal ends	Small and placed relatively closely to one another	Not documented	Not documented	Not documented
References	This study	Krammer [6]; Leira et al. [30]	Tremarin et al. [31]; Hirota & Ohtsuka [32]; Metzeltin & Lange-Bertalot [33]; Pereira et al. [34]	Krammer [6]; Serieyssol [35]	Krammer [6]; Patrick & Reimer [36]; Krammer & Lange-Bertalot [37];

**Table 4:** Morphological delineation of *Pinnularia seouloflexuosa* sp. nov. in comparison with other large-celled linear *Pinnularia* species.

Characters	*P. seouloflexuosa* sp. nov.	*P. baicalflexuosa*	*P. streptoraphe*	*P. spinifera*	*P. nobilis* var. *regularis*
Length (µm)	147–151	109–116	134–174	130–215	200–270
Width (µm)	19–21	17.5–19	24–27	20–25	32.5–39
Length/Width ratio	7.01–7.66	Not documented	Not documented	6.0–8.6	Not documented
Striae density (/10 µm)	7–9	7–8	5	6–8	5
Alveolus	6	5–6	Not documented.	6–7	Not documented.
Striae pattern	Radiate at center, parallel-convergent at poles	Radiate at center, convergent at ends	Mostly parallel, slightly radiate at center	Weakly radiate at center, parallel at apices	Radiate at center, convergent at apices
Valve outline	Linear, slight central protrusion, concave inward	Linear with parallel sides	Linear with broad, parallel margins	Linear, straight, or slightly concave sides	Linear, slightly swollen central valve
Apices (ends)	Rounded	Broadly rounded	Rounded	Broadly rounded	Slightly swollen and rounded
Central nodule	Visible, supporting structure	Not documented	Visible, thick silica cell wall	Visible, distinct	Visible, slightly asymmetric
Raphe	Lateral, complex; large central pores, terminal fissures hooked	Semicomplex, undulate	Complex, prominent features	Lateral, weakly sinuous, question-mark-shaped terminal fissures	Lateral, complex; proximal ends have large central pores, terminal fissures hooked
Short spines	Absent	Not documented	Not documented	Present on valve face-mantle junction	Not documented
Axial area	Linear, occupying 1/3–1/4 of valve width	Linear, narrow, tapering at ends	Linear, tapering at ends, <1/3 width of valve	Linear, 1/3–1/5 of valve width	Slightly asymmetric, closer to secondary side
Central area	Elliptic, asymmetric, broader on primary side	Small, asymmetrically elliptic	Small, asymmetric, expanded on primary side	Elliptic, asymmetrical, broader on primary side	Elliptic, asymmetric, broader on primary side
References	This study	Kulikovskiy et al. [8]	Krammer [6]; Patrick & Reimer [36]; Krammer & Lange-Bertalot [37];	Potapova et al. [38]	Krammer [6]

**Table 5:** Comparative morphology of *Pinnularia paristriata* sp. nov. and related species exhibiting linear to linear-elliptic valve shapes.

Characters	*P. paristriata* sp. nov.	*P. neomajor*	*P. neglectiformis*	*P. viridiformis*	*P. viridis*
Length (µm)	57.6–75.1	114–250	80–130	67–145	100–182
Width (µm)	13.8–16.5	17–30	16–20	14–21	21–30
L/W ratio	3.7–4.6	7.3–9	5–6.5	3.8–7.5	5–6.5
Striae/10 µm	10–11	6–8	8–9	7–9	6–7
Alveolus	6–7	Not documented	Not documented	Not documented	Not documented
Outline	linear-elliptic, parallel or slightly convex	linear, sometimes slightly swollen in the central and terminal portion	linear, sides parallel, slightly convex or triundulate	linear, sometimes weakly convex	linear, sides parallel or slightly convex or triundulate
Apices	rounded	rounded	rounded to cuneiform rounded	broadly rounded	rounded
Striae	mostly parallel, occasionally weakly radiate in the center and slightly convergent at the apices, with minimal angular variation	radiate in the middle, slightly convergent towards the ends	moderately radiate in the middle, slightly convergent towards the ends	slightly to moderately radiate in the middle, parallel to slightly convergent towards the ends	moderately radiate in the middle, slightly convergent towards the ends
Raphe	compound and markedly curved	lateral with moderately undulated outer fissure	semicomplex outer fissure undulate	compound, noticeably crooked	broadly lateral to semicomplex, than outer fissures undulate
Axial Area	1/5–1/4 the breadth of the valve, linear, tapering on the ends	linear, moderately wide, tapering towards the ends, 1/4–1/3 of the valve breadth	1/5–1/4 the breadth of the valve, linear or tapering lanceolate to the ends	1/5–1/4 the breadth of the valve, linear, tapering on the ends	1/5–1/4 the breadth of the valve, linear, tapering lanceolate at the ends
Central area	slightly expanded, irregular, and asymmetrical	slightly expanding, irregular, and frequently asymmetrical	roundish or irregular, a little wider than the axial area, almost asymmetrical	roundish, a little wider than the axial area, almost asymmetrical	variable, roundish or irregular, a little wider than the axial area, almost asymmetrical
Proximal raphe ends	bent to one side, central pores small, round	bent to one side, the central pores round and close together	bent to one side, central pores small, round	bent to one side, central pores small, round, close standing	bent to one side, central pores small, round
Terminal raphe fissure	shaped like question marks	widely hooked	viridis-like	viridis-like	viridis-like
References	This study	Krammer [39]	Krammer [6]	Sonneman [28]; Krammer [39]	Krammer [6]

**Table 6:** Estimates of evolutionary divergence among 20 closely related *Pinnularia* species based on SSU rRNA (1678 bp).

	Species	Strains	1	2	3	4	5	6	7	8	9	10	11	12	13	14	15	16	17	18	19	20
1	*Pinnularia latocentra*	PQ034657		0.003	0.006	0.007	0.002	0.005	0.002	0.002	0.006	0.007	0.004	0.001	0.005	0.005	0.003	0.003	0.004	0.005	0.006	0.006
2	*Pinnularia rhombocentra*	PQ034660	0.017		0.006	0.007	0.003	0.005	0.003	0.003	0.006	0.007	0.004	0.003	0.005	0.005	0.003	0.002	0.005	0.005	0.006	0.007
3	*Pinnularia seouloflexuosa*	PP940033	0.051	0.060		0.006	0.006	0.007	0.006	0.006	0.004	0.006	0.006	0.006	0.008	0.005	0.006	0.006	0.006	0.007	0.004	0.005
4	*Pinnularia paristriata*	PV953636	0.069	0.076	0.054		0.007	0.008	0.007	0.007	0.006	0.002	0.007	0.007	0.009	0.007	0.007	0.007	0.008	0.008	0.006	0.007
5	*Pinnularia anglica*	AM501980	0.005	0.017	0.053	0.071		0.005	0.002	0.002	0.006	0.007	0.004	0.001	0.005	0.005	0.002	0.003	0.005	0.005	0.006	0.006
6	*Pinnularia* cf. *gibba*	EF151977	0.033	0.037	0.067	0.085	0.036		0.005	0.005	0.007	0.007	0.004	0.005	0.002	0.006	0.005	0.005	0.003	0.003	0.007	0.007
7	*Pinnularia* cf. *interrupta*	AJ544658	0.004	0.017	0.054	0.072	0.004	0.034		0.002	0.006	0.007	0.004	0.002	0.005	0.005	0.002	0.004	0.005	0.005	0.006	0.006
8	*Pinnularia grunowii*	JN418588	0.006	0.017	0.052	0.071	0.005	0.034	0.005		0.006	0.007	0.004	0.002	0.005	0.005	0.003	0.004	0.005	0.005	0.006	0.006
9	*Pinnularia neglectiformis*	JN418596	0.050	0.057	0.026	0.056	0.051	0.066	0.054	0.050		0.006	0.006	0.006	0.008	0.005	0.006	0.006	0.007	0.007	0.003	0.005
10	*Pinnularia neomajor*	JN418571	0.071	0.077	0.055	0.009	0.071	0.084	0.072	0.071	0.058		0.006	0.007	0.008	0.007	0.007	0.007	0.007	0.008	0.006	0.007
11	*Pinnularia nodosa*	JN418587	0.021	0.026	0.051	0.063	0.023	0.031	0.022	0.024	0.050	0.061		0.004	0.005	0.005	0.004	0.004	0.004	0.005	0.006	0.006
12	*Pinnularia obscura*	AM501986	0.003	0.017	0.051	0.070	0.004	0.033	0.004	0.005	0.051	0.070	0.021		0.005	0.005	0.002	0.003	0.005	0.005	0.006	0.006
13	*Pinnularia parvulissima*	JN418591	0.039	0.040	0.075	0.091	0.040	0.006	0.037	0.039	0.076	0.087	0.035	0.039		0.006	0.005	0.006	0.003	0.004	0.008	0.008
14	*Pinnularia* sp.	JN418572	0.035	0.041	0.043	0.072	0.038	0.050	0.038	0.038	0.042	0.074	0.041	0.035	0.052		0.005	0.005	0.006	0.006	0.005	0.006
15	*Pinnularia* sp.	JN418580	0.010	0.018	0.054	0.071	0.009	0.038	0.009	0.010	0.053	0.071	0.022	0.010	0.042	0.040		0.004	0.005	0.005	0.006	0.006
16	*Pinnularia subanglica*	PP065733	0.017	0.009	0.062	0.077	0.018	0.035	0.018	0.019	0.058	0.077	0.027	0.015	0.042	0.043	0.021		0.005	0.005	0.006	0.007
17	*Pinnularia subcapitata*	AM501979	0.031	0.037	0.063	0.084	0.034	0.012	0.033	0.034	0.063	0.081	0.029	0.032	0.014	0.049	0.037	0.036		0.003	0.007	0.007
18	*Pinnularia subgibba*	KT072984	0.041	0.044	0.068	0.086	0.042	0.016	0.043	0.043	0.070	0.082	0.035	0.040	0.017	0.056	0.045	0.040	0.015		0.007	0.007
19	*Pinnularia viridiformis*	AM501985	0.053	0.059	0.031	0.057	0.054	0.070	0.055	0.053	0.017	0.059	0.053	0.052	0.080	0.043	0.056	0.059	0.066	0.074		0.005
20	*Pinnularia viridis*	AM502023	0.067	0.075	0.047	0.072	0.070	0.078	0.067	0.067	0.044	0.070	0.065	0.068	0.085	0.063	0.070	0.074	0.075	0.084	0.047	

**Table 7:** Estimates of evolutionary divergence among 20 closely related *Pinnularia* species based on *rbcL* (752 bp).

	Species	Strains	1	2	3	4	5	6	7	8	9	10	11	12	13	14	15	16	17	18	19	20
1	*Pinnularia latocentra*	PQ044335		0.009	0.010	0.011	0.010	0.007	0.005	0.010	0.011	0.008	0.005	0.010	0.009	0.009	0.009	0.009	0.009	0.012	0.010	0.010
2	*Pinnularia rhombocentra*	PQ044336	0.050		0.009	0.010	0.008	0.008	0.009	0.009	0.010	0.008	0.009	0.008	0.008	0.009	0.006	0.007	0.007	0.009	0.009	0.009
3	*Pinnularia seouloflexuosa*	PP962248	0.057	0.050		0.006	0.008	0.009	0.009	0.004	0.007	0.008	0.010	0.009	0.008	0.002	0.009	0.008	0.009	0.009	0.005	0.005
4	*Pinnularia paristriata*	PV956143	0.071	0.066	0.023		0.010	0.011	0.011	0.005	0.005	0.010	0.011	0.010	0.010	0.006	0.010	0.009	0.010	0.010	0.007	0.006
5	*Pinnularia* cf. *gibba*	EF143304	0.059	0.039	0.043	0.056		0.007	0.009	0.008	0.010	0.007	0.009	0.003	0.006	0.008	0.007	0.005	0.006	0.006	0.009	0.008
6	*Pinnularia grunowii*	JN418658	0.032	0.043	0.053	0.068	0.034		0.007	0.009	0.011	0.007	0.007	0.007	0.008	0.009	0.008	0.008	0.008	0.010	0.010	0.010
7	*Pinnularia microfrauenbergiana*	KM349979	0.015	0.048	0.053	0.062	0.055	0.034		0.010	0.010	0.008	0.005	0.009	0.009	0.009	0.009	0.009	0.009	0.011	0.011	0.010
8	*Pinnularia neglectiformis*	MH707959	0.055	0.050	0.012	0.019	0.039	0.055	0.055		0.006	0.008	0.009	0.008	0.008	0.004	0.009	0.007	0.008	0.008	0.004	0.003
9	*Pinnularia neomajor*	JN418641	0.069	0.066	0.030	0.012	0.059	0.073	0.064	0.022		0.010	0.011	0.010	0.010	0.006	0.010	0.009	0.010	0.010	0.006	0.005
10	*Pinnularia nodosa*	JN418657	0.043	0.041	0.041	0.056	0.030	0.032	0.046	0.039	0.055		0.008	0.007	0.007	0.008	0.007	0.006	0.007	0.007	0.009	0.008
11	*Pinnularia obscura*	AM710452	0.018	0.051	0.057	0.068	0.050	0.029	0.013	0.055	0.069	0.041		0.009	0.009	0.010	0.008	0.008	0.008	0.011	0.010	0.010
12	*Pinnularia parvulissima*	JN418661	0.057	0.045	0.044	0.058	0.005	0.032	0.053	0.041	0.060	0.032	0.048		0.005	0.008	0.008	0.005	0.006	0.006	0.009	0.009
13	*Pinnularia siberiosinistra*	KM349978	0.057	0.043	0.046	0.060	0.020	0.041	0.053	0.043	0.059	0.029	0.048	0.018		0.008	0.007	0.004	0.005	0.006	0.009	0.008
14	*Pinnularia streptoraphe*	KM349984	0.055	0.048	0.002	0.023	0.041	0.051	0.051	0.010	0.029	0.039	0.055	0.043	0.044		0.009	0.008	0.008	0.009	0.005	0.004
15	*Pinnularia subanglica*	JN418668	0.050	0.022	0.050	0.064	0.030	0.037	0.048	0.046	0.062	0.034	0.046	0.036	0.032	0.048		0.005	0.007	0.006	0.009	0.009
16	*Pinnularia subcapitata*	AM710445	0.053	0.036	0.041	0.054	0.013	0.037	0.050	0.037	0.053	0.020	0.048	0.015	0.012	0.039	0.020		0.005	0.002	0.008	0.008
17	*Pinnularia subgibba*	KT072931	0.051	0.037	0.046	0.058	0.020	0.037	0.051	0.043	0.057	0.030	0.046	0.018	0.017	0.044	0.036	0.017		0.007	0.009	0.008
18	*Pinnularia vietnamogibba*	OL704399	0.064	0.039	0.039	0.052	0.017	0.046	0.060	0.035	0.050	0.024	0.057	0.019	0.017	0.037	0.022	0.002	0.024		0.009	0.009
19	*Pinnularia viridiformis*	AM710451	0.060	0.055	0.017	0.029	0.048	0.064	0.064	0.008	0.025	0.044	0.064	0.050	0.048	0.015	0.048	0.043	0.048	0.042		0.003
20	*Pinnularia viridis*	AM710490	0.060	0.050	0.015	0.023	0.046	0.061	0.060	0.007	0.020	0.043	0.060	0.048	0.046	0.013	0.050	0.041	0.044	0.039	0.008	

## Data Availability

The data supporting the findings of this study are available from the corresponding author upon reasonable request.

## Supplementary Materials

The following supporting information can be downloaded at: https://www.mdpi.com/article/10.3390/plants14203206/s1. Figure S1: Secondary structure model of 18S rRNA (Clade B) in *Pinnularia* species, inferred from phylogenetic relationships shown in Figure 10 of the main text. Figure S2: Secondary structure model of 18S rRNA (Clade C) in *Pinnularia* species, corresponding to phylogenetic Clade C shown in Figure 10. Figure S3: Secondary structure model of rbcL (Clade B) in *Pinnularia* species, based on the phylogenetic analysis in Figure 11. Figure S4: Secondary structure model of rbcL (Clade C) in *Pinnularia* species, inferred from the rbcL phylogeny shown in Figure 11. Table S1: Strains and GenBank accession numbers used for phylogenetic analyses based on SSU rRNA and rbcL genes.

## Abbreviations

DN	Deposition Number (type material at KCTC)
DO	Dissolved oxygen (mg L^−1^)
EC	Electrical conductivity (µS cm^−1^)
GPS	Global Positioning System (latitude, longitude in decimal degrees)
HYU	Hanyang University (Seoul, Korea)
KCTC	Korean Collection for Type Cultures (KRIBB, Jeongeup, Korea)
LM	Light microscopy
ML	Maximum likelihood
NCBI	National Center for Biotechnology Information (USA)
NIBR	National Institute of Biological Resources (Incheon, Korea)
NTU	Nephelometric Turbidity Unit
*rbcL*	Ribulose-1,5-bisphosphate carboxylase/oxygenase large subunit gene
SEM	Scanning electron microscopy
SSU rDNA	Small subunit ribosomal DNA
Turb	Turbidity (NTU)
WT	Water temperature (°C)
