# Supplementary material for: Four New Pinnularia Ehrenberg Species (Bacillariophyta) from Urban Freshwater Streams in South Korea
Source: Plants (Basel). 2025 Oct 18;14(20):3206. doi: 10.3390/plants14203206 (PMC12567537; doi:10.3390/plants14203206)

Supplementary Material

Four New *Pinnularia* Species (Bacillariophyta) from Urban Freshwater Streams in South Korea

Yuyao Li [1], Weihan Wang [2], Byeong-Hun Han [1,2], Su-Ok Hwang [3], Baik-Ho Kim [1,3,4,*]

[1] Department of Environmental Science, Hanyang University, Seoul, Republic of Korea

[2] Dongmoon ENT, Guro-gu, Seoul 08377, Republic of Korea

[3] Research Institute for Natural Sciences, Hanyang University, Seoul, Republic of Korea

[4] Department of Life Science, Hanyang University, Seoul, Republic of Korea

*Corresponding author: B.-H. Kim (Email: tigerk@hanyang.ac.kr)

Supplementary Table S1. Strains and GenBank accession numbers used for phylogenetic analyses based on SSU rRNA and rbcL genes.

No	Taxon (SSU rRNA)	Strain	Accession number	Taxon (rbcL gene)	Strain	Accession number
1	*Pinnularia latocentra*	HYU-D117	PQ034657	*Pinnularia latocentra*	HYU-D117	PQ044335
2	*Pinnularia latocentra*	HYU-D127	PQ178304	*Pinnularia latocentra*	HYU-D127	PQ168579
3	*Pinnularia latocentra*	HYU-D128	PQ178305	*Pinnularia latocentra*	HYU-D128	PQ168580
4	*Pinnularia rhombocentra*	HYU-D118	PQ034660	*Pinnularia rhombocentra*	HYU-D118	PQ044336
5	*Pinnularia rhombocentra*	HYU-D129	PQ178307	*Pinnularia rhombocentra*	HYU-D129	PQ168581
6	*Pinnularia rhombocentra*	HYU-D130	PQ178454	*Pinnularia rhombocentra*	HYU-D130	PQ168582
7	*Pinnularia seouloflexuosa*	HYU-D106	PP940033	*Pinnularia seouloflexuosa*	HYU-D106	PP962248
8	*Pinnularia seouloflexuosa*	HYU-D109	PP949575	*Pinnularia seouloflexuosa*	HYU-D109	PP962249
9	*Pinnularia seouloflexuosa*	HYU-D110	PP949576	*Pinnularia seouloflexuosa*	HYU-D110	PP962250
10	*Pinnularia paristriata*	HYU-D141	PV953636	*Pinnularia paristriata*	HYU-D141	PV956143
11	*Pinnularia paristriata*	HYU-D144	PV953671	*Pinnularia paristriata*	HYU-D144	PV956146
12	*Pinnularia paristriata*	HYU-D145	PV953673	*Pinnularia paristriata*	HYU-D145	PV956147
13	*Pinnularia acuminata*	Pin 876 TM	JN418597	*Pinnularia acrosphaeria*	TCC472	KC736612
14	*Pinnularia anglica*	AT-100Gel01	AM501980	*Pinnularia acuminata*	Pin 876 TM	JN418667
15	*Pinnularia borealis var. subislandica*	(Tor3)a	JN418575	*Pinnularia australogibba*	12 CS-2011	JN418673
16	*Pinnularia brebissonii*	UTEX FD274	HQ912604	*Pinnularia baicalflexuosa*	B054-3	KM349984
17	*Pinnularia cf. gibba*	12	EF151977	*Pinnularia baicalodivergens*	B112	KM349992
18	*Pinnularia cf. interrupta*	TE1	AJ544658	*Pinnularia borealis*	Alka 1	JN418662
19	*Pinnularia cf. marchica*	(Ecrins4)a	JN418569	*Pinnularia borealis cf. var.subislandica*	(Tor12)d	JN418640
20	*Pinnularia cf. microstauron*	(B2)c	JN418568	*Pinnularia borealis var. subislandica*	(Tor3)a	JN418645
21	*Pinnularia grunowii*	Pin 889 MG	JN418588	*Pinnularia brebissonii*	UTEX FD274	HQ912468
22	*Pinnularia mesolepta*	AT-160Gel30	AM501994	*Pinnularia cf. gibba*	12	EF143304
23	*Pinnularia mesolepta*	AT-161.05	AM502024	*Pinnularia cf. marchica*	(Ecrins4)a	JN418639
24	*Pinnularia microstauron*	AT-105Gel08	AM501981	*Pinnularia divergens*	D31_023	KM350034
25	*Pinnularia neglectiformis*	Pin 706 F	JN418596	*Pinnularia grunowii*	Pin 889 MG	JN418658
26	*Pinnularia neglectiformis*	CBac2019016	OL780283	*Pinnularia lacustrigibba*	PIN19Cra	MH670406
27	*Pinnularia neomajor*	(Tor1)a	JN418571	*Pinnularia mesolepta*	AT-160Gel30	AM710461
28	*Pinnularia nodosa*	Pin 885 TM	JN418587	*Pinnularia microfrauenbergiana*	B025	KM349979
29	*Pinnularia obscura*	AT-70Gel12b	AM501986	*Pinnularia microgibba*	VP289	OL704398
30	*Pinnularia parvulissima*	Pin 877 TM	JN418591	*Pinnularia minigibba*	VP284	OL704397
31	*Pinnularia rupestris*	PRUP1	AJ867027	*Pinnularia ministomatophora*	VP563	OL704402
32	*Pinnularia rupestris*	AT-160Gel10	AM501992	*Pinnularia neglectiformis*	Pin 706 F	MH707959
33	*Pinnularia sp.*	1 CS-2011	JN418572	*Pinnularia neomajor*	(Tor1)a	JN418641
34	*Pinnularia sp.*	5 CS-2011	JN418580	*Pinnularia neomajor var. inflata*	B168	KM349997
35	*Pinnularia sp.*	7 CS-2011	JN418582	*Pinnularia nodosa*	Pin 885 TM	JN418657
36	*Pinnularia subanglica*	Pin 650 K	JN418598	*Pinnularia obscura*	AT-70Gel12b	AM710452
37	*Pinnularia subanglica*	HYU-D091	PP065733	*Pinnularia paradubitabilis*	VP236	OL704395
38	*Pinnularia subcapitata*	AT-100.01	AM501979	*Pinnularia parvulissima*	B028	KM349982
39	*Pinnularia subcapitata var. elongata*	(Wie)c	JN418579	*Pinnularia parvulissima*	Pin 877 TM	JN418661
40	*Pinnularia subcommutata var. nonfasciata*	Corsea 10	JN418584	*Pinnularia septentrionalis*	B147	KM349995

41	*Pinnularia subgibba*	TCC608	KT072984	*Pinnularia shivae*	VN399	KM350050
42	*Pinnularia substreptoraphe*	AT-70.09	AM502036	*Pinnularia siberiosinistra*	B024-1	KM349978
43	*Pinnularia termitina*	L1662	AM743107	*Pinnularia sp.*	5 CS-2015	JN418650
44	*Pinnularia termitina*	UTEX FD484	HQ912601	*Pinnularia stomatophora*	D11_014	KM350019
45	*Pinnularia viridiformis*	AT-70.10	AM501985	*Pinnularia subanglica*	Pin 650 K	JN418668
46	*Pinnularia viridiformis*	(Enc2)a	JN418574	*Pinnularia subcapitata*	AT-100.01	AM710445
47	*Pinnularia viridiformis*	Pin 870 MG	JN418589	*Pinnularia subcommutata*	Corsea10	MH707982
48	*Pinnularia viridis*	AT-161.02	AM502023	*Pinnularia subgibba*	TCC608	KT072931
49				*Pinnularia termitina*	UTEX FD484	HQ912465
50				*Pinnularia vietnamogibba*	VP290	OL704399
51				*Pinnularia viridiformis*	AT-70.10	AM710451
52				*Pinnularia viridiformis*	Pin 870 MG	JN418659
53				*Pinnularia viridis*	AT-161.02	AM710490
1	*Caloneis lauta*	AT-160Gel04	AM502039	*Caloneis lauta*	AT-160Gel04	AM710506
2	*Caloneis lewisii*	UTEX FD54	HQ912580	*Fragilaria bidens*	HYU-D133	PQ168585
3	*Craticula importuna*	HYU-D135	PQ176818	*Navicula symmetrica*	HYU-D072	OR880639
4	*Craticula subminuscula*	HYU-D136	PQ176820	*Navicula veneta*	HYU-D089	OR880638
5	*Fragilaria bidens*	HYU-D133	PQ176815	*Navicula viridula var. rostellata*	HYU-D124	PQ044339
6	*Navicula symmetrica*	HYU-D072	OR841389	*Nitzschia acicularis*	HYU-D134	PQ550011
7	*Navicula viridula var. rostellata*	HYU-D124	PQ034684	*Nitzschia palea*	HYU-D070	OR880636
8	*Nitzschia acicularis*	HYU-D134	PQ176817	*Sellaphora blackfordensis*	(Bfp04)02	JN418674
9	*Nitzschia palea*	HYU-D070	OR841379	*Sellaphora laevissima*	THR3	EF143313
10	*Sellaphora blackfordensis*	(Bfp5x8)F1-3	JN418599	*Sellaphora pupula*	THR15	EF143321
11	*Sellaphora laevissima*	THR4	EF151981			
12	*Sellaphora pupula*	THR14	EF151963			

Supplementary Figure S1. Secondary structure model of 18S rRNA (Clade B) in *Pinnularia* species inferred from the phylogenetic relationships shown in Figure 10 of the main text. This structure compares newly described species with their closest phylogenetic relatives.

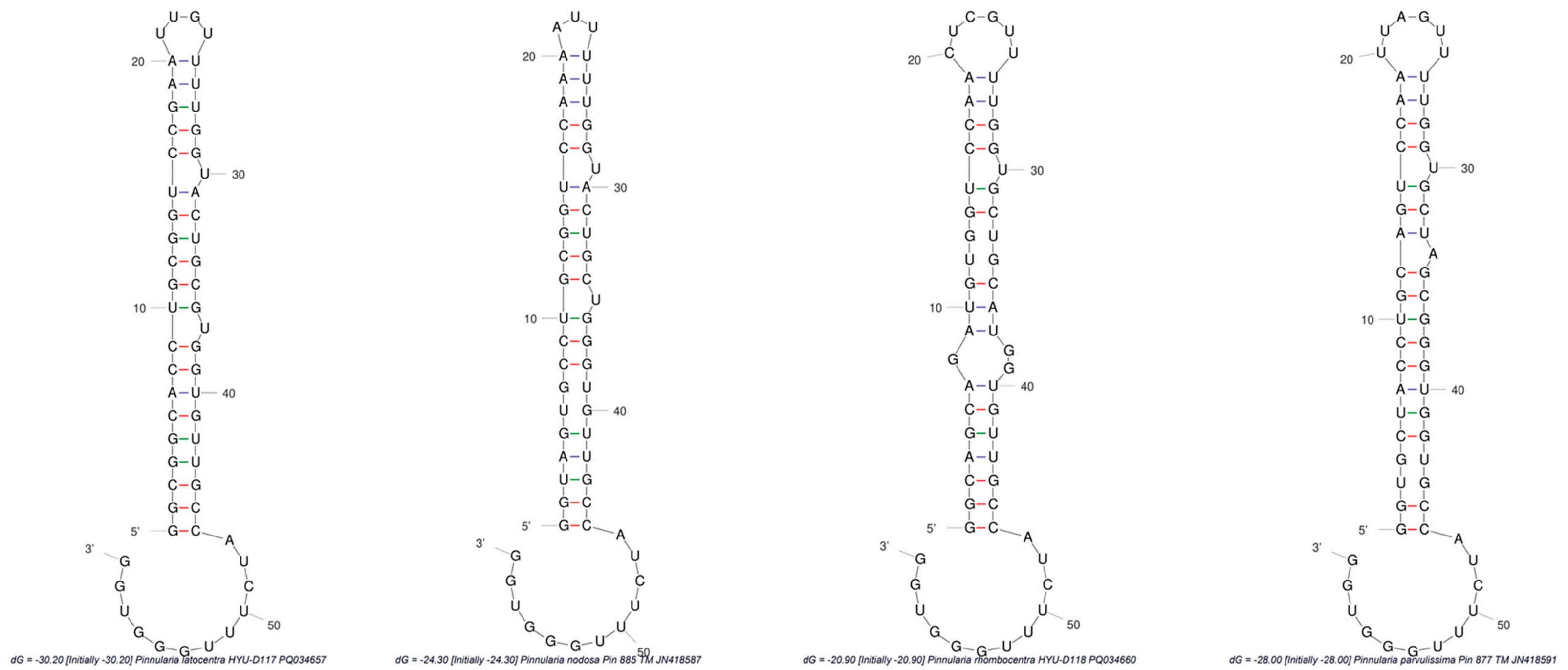

Supplementary Figure S2. Secondary structure model of 18S rRNA (Clade C) in *Pinnularia* species corresponding to the phylogenetic Clade C shown in Figure 10. This model highlights the shared and divergent structural features among related taxa.

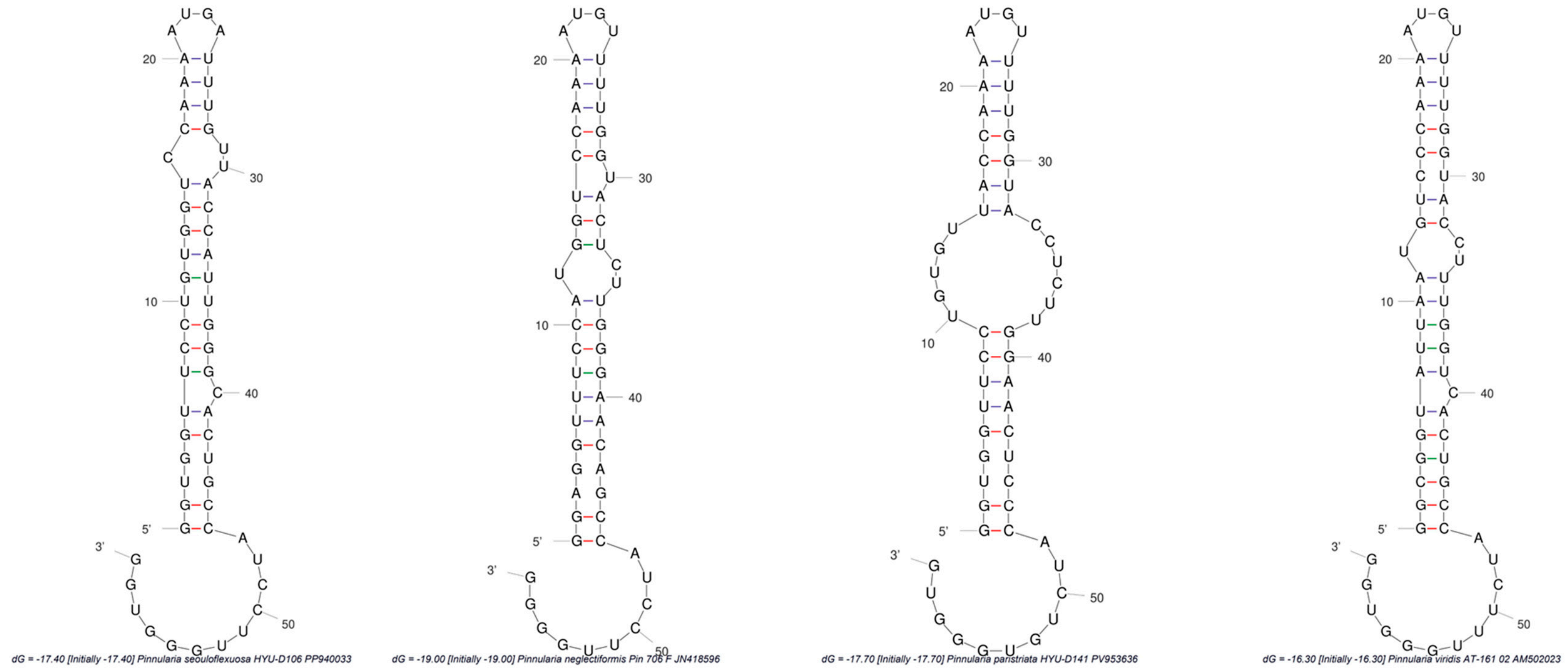

Supplementary Figure S3. Secondary structure model of rbcL (Clade B) in *Pinnularia* species. Based on Figure 11, this model illustrates the structural variation between the newly described taxa and their phylogenetic neighbors.

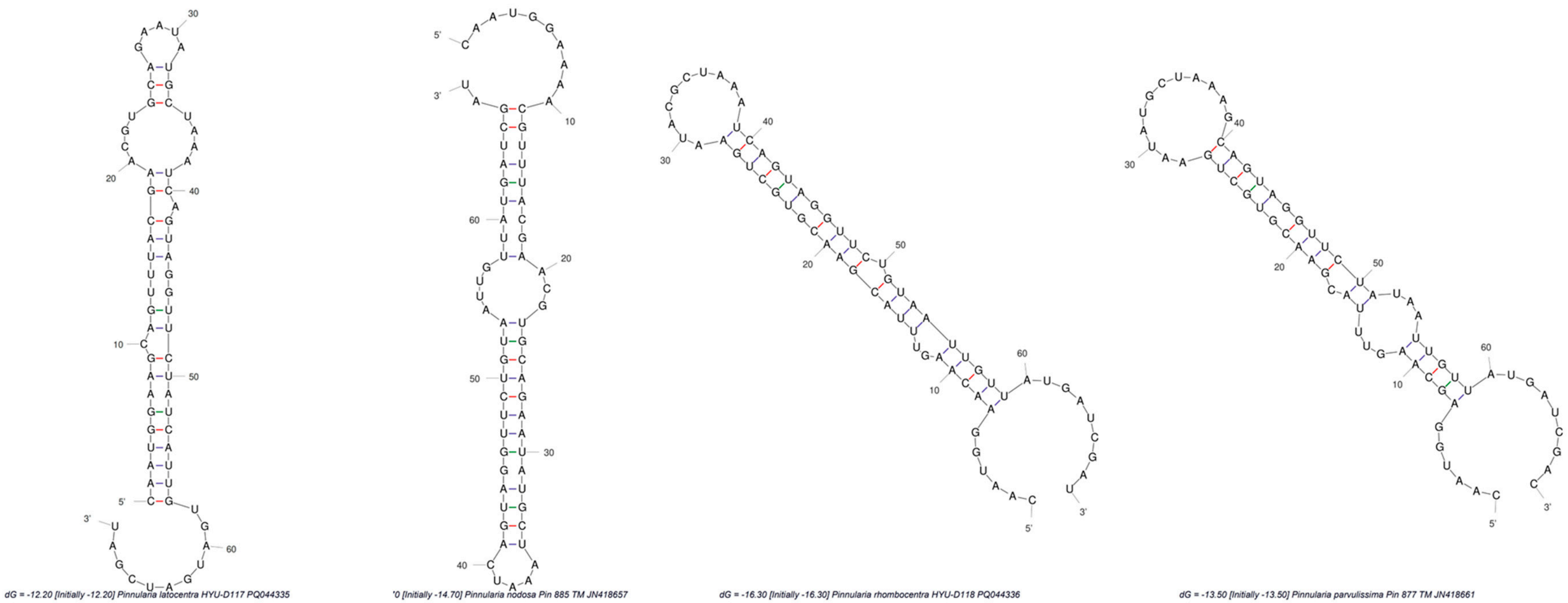

Supplementary Figure S4. Secondary structure model of rbcL (Clade C) in *Pinnularia* species. As inferred from the rbcL phylogeny (Figure 11), this model highlights the structural differences between the novel species and their closest clade members.

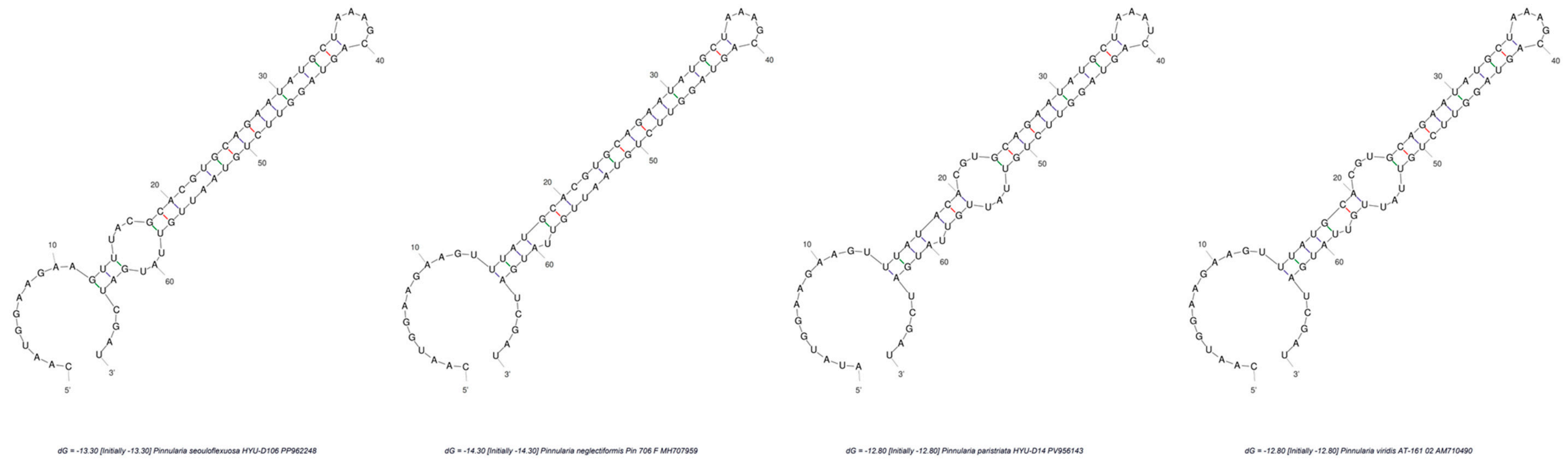

Supplement: Supplementary file 1 [file plants-14-03206-s001.zip › plants-3845124-supplementary.pdf]
